# Acquisition of antibodies that block *Plasmodium falciparum* adhesion to placental receptor chondroitin sulfate A with increasing gravidity in Malian women

**DOI:** 10.3389/fimmu.2023.1330962

**Published:** 2024-01-11

**Authors:** Almahamoudou Mahamar, Moussa Traore, Bruce Swihart, Oumar Attaher, Bacary Soumana Diarra, Gaoussou Santara, Djibrilla Issiaka, Amadou Barry, Youssoufa Sidibé, Yahia T. Dicko, Sekouba Keita, Oulematou Ndiaye, Alassane Dicko, Patrick E. Duffy, Michal Fried

**Affiliations:** ^1^ Malaria Research & Training Center, Faculty of Medicine, Pharmacy and Dentistry, University of Sciences Techniques and Technologies of Bamako, Bamako, Mali; ^2^ Biostatistics Research Branch, National Institute of Allergy and Infectious Diseases, NIH, Rockville, MD, United States; ^3^ Laboratory of Malaria Immunology and Vaccinology, National Institute of Allergy and Infectious Diseases, NIH, Bethesda, MD, United States

**Keywords:** placental malaria, infected erythrocyte adhesion, chondroitin sulfate A, anti-adhesion antibodies, small for gestational age

## Abstract

In malaria-endemic areas, pregnant women are more susceptible to *Plasmodium falciparum* infection, especially primigravidae. During pregnancy, parasites sequester in the placenta and bind to the receptor chondroitin sulfate (CSA). This unique adhesion is mediated by the parasite protein VAR2CSA expressed on the surface of infected erythrocytes (IE). Placental malaria is associated with poor pregnancy outcomes including perinatal mortality, preterm delivery, small for gestational age (SGA) and low birthweight deliveries. Over successive pregnancies, women acquire functional antibodies that inhibit IE adhesion to CSA. Here, we examine the development of anti-adhesion activity and the breadth of anti-adhesion activity as a function of number of previous pregnancies, using samples collected from pregnant women living in an area with high seasonal malaria transmission. Women reached plateau levels of anti-adhesion activity and breadth of anti-adhesion activity after 5 pregnancies. We related the level of anti-adhesion activity and reactivity with surface IE to SGA 19/232 pregnancies resulted in SGA, and report that an increase of 10% in median anti-adhesion activity reduced the odds of SGA by 13% and this relationship approached significance. Further, at an anti-adhesion activity level of 43.7%, an increase of 10% in the breadth of activity significantly reduced the odds of SGA by 21.5%. Antibodies that recognize IE surface increased over successive pregnancies, but were not associated with a reduction in SGA. These results can serve as a guideline for assessing vaccine candidates aiming to reduce poor pregnancy outcomes associated with placental malaria.

## Introduction

Malaria during pregnancy has a unique epidemiological presentation: susceptibility is highest during the first pregnancy and diminishes over successive pregnancies (1). Malaria during pregnancy is associated with poor outcomes for both the mother and her newborn child: maternal anemia, low birthweight deliveries, stillbirth, early neonatal death, preterm delivery (PTD), and small for gestational age (SGA) (2–5). In a longitudinal cohort of pregnant women conducted in Mali, we described that malaria infection increased the risk of stillbirth and PTD in primigravidae, as well as risk of early neonatal death in secundigravidae and multigravidae (6). Although malaria infection increased the risk of SGA, the relationships were not significant (6). We speculate that SGA may be associated with a more chronic subpatent infection in this population that is often difficult to detect, while anti-adhesion antibodies may play an important role in modifying the risk of SGA.

During pregnancy, *Plasmodium falciparum* parasites sequester in the placenta and bind to the receptor chondroitin sulfate A (CSA) expressed on the syncytiotrophoblast surface (7). Parasite adhesion to CSA is mediated by VAR2CSA, a member of the *P. falciparum* erythrocyte membrane protein 1 (PfEMP1) variant antigen family (8–10). Multiple studies have evaluated acquisition of immunity to placental parasites using different approaches, such as measuring levels of neutralizing antibodies that can block parasite adhesion to CSA, antibody reactivity with the surface of infected erythrocytes (IE) by flow cytometry, IE agglutination, and IE opsonizing activity. All studies uniformly observed parity-dependent acquisition of antibodies to placental parasites or laboratory isolates selected to bind CSA [reviewed in (11)], with significantly higher antibody levels in multigravidae compared to primigravidae. Similar observations were made using parasites or plasma samples collected in multiple sites in sub-Saharan Africa and South-East Asia. In western Kenya where malaria transmission is intense, we found that birthweight was 398g greater when maternal sera reduced parasite adhesion to CSA by ≥65%, versus sera without this level of activity (12). Naturally acquired maternal antibodies reduced adhesion to CSA of placental IE collected at various geographic locations (13), suggesting the antibodies target a conserved epitope across VAR2CSA allelic forms.

In recent decades, multiple groups have pursued development of a pregnancy malaria vaccine to prevent poor pregnancy outcomes related to malaria infection. However, vaccine design based on the large, cysteine-rich, highly variant VAR2CSA protein has been challenging, and candidates studied to date have mainly elicited antibodies with homologous but not heterologous anti-adhesion activity (14). In the current study, we examined plasma collected from pregnant women in an area with intense seasonal malaria transmission to further characterize acquisition of natural immunity to placental parasites in order to guide future vaccine design. We define full functional activity by measuring anti-adhesion antibodies over successive pregnancies, and relate these with pregnancy outcomes, specifically SGA.

## Methods

### Human subjects

Pregnant women in this study were enrolled into a longitudinal cohort study of mother-infant pairs as well as a cross-sectional study in Ouélessébougou, Mali. Written informed consent was obtained from study participants or parents/guardians of pregnant adolescents after receiving written and oral explanation from a study clinician in their native language. Study protocols and procedures were approved by the Institutional Review Boards at the National Institute of Allergy and Infectious Diseases, National Institutes of Health, and by the Ethics Committee of the Faculty of Medicine, Pharmacy and Dentistry, University of Bamako, Bamako, Mali. The protocols are registered at Clinicaltrials.gov under identifiers NCT01168271 and NCT02471378.

### Parasite and plasma sample selection

232 plasma samples were collected at delivery from women enrolled during pregnancy into a longitudinal cohort study of mother infant pairs (6). The 232 plasma samples were tested in multiple batches. Of the 232 plasma samples, 161 randomly selected based on parasite material availability were also used to measure reactivity with surface IE. Fewer samples were used for IE surface recognition assays due to limitations in the amount of fresh parasite isolates available prioritizing anti-adhesion activity assays. Parasites used in the assays were collected from pregnant women participating in a cross-sectional study that enrolled pregnant women during antenatal clinic visit. Only fresh heterologous parasite isolates collected from pregnant women were used to measure levels and the breadth of anti-adhesion activity and surface reactivity.

Plasma samples were selected based on the number of previous pregnancies in the multigravidae group, such that the proportion of samples in women with 2 or more previous pregnancies was similar to that in the full cohort. The percentage of women by number of previous pregnancies in the full set and the subset used here is as follows: 2 previous pregnancies, 14.3% and 12.5%; 3 previous pregnancies, 11.6% and 15.9%; 4 previous pregnancies, 9.4% and 10.8%; 5 previous pregnancies, 6.5% and 10.8%; 6 previous pregnancies, 6.3% and 9.1%; 7 previous pregnancies, 4.2% and 9.1%; >=8 previous pregnancies, 4.8% and 5.2%. To reduce potential background reactivity in samples collected during acute infection, preference was made to include samples from women that were not infected at delivery.

### Binding inhibition assay

Peripheral blood samples were collected from malaria-infected pregnant women presenting for antenatal clinical visit. Ring stage parasite samples were allowed to mature to trophozoite/schizont stages during *in vitro* culture for 16–24 h after sample collection. A total of 42 parasite isolates were used in the anti-adhesion assays collected over a period of about 7 months. Mature parasite forms were enriched to 2-20% parasitemia by gelatin flotation method. 20 μl CSA from bovine trachea (Sigma) at a concentration of 20 μg/ml was immobilized by adsorption on Petri dishes. The parasite suspension was preincubated with plasma diluted 1:5 for 30 min at room temperature, then allowed to bind to the immobilized receptor for 30 min at room temperature. Plates were washed with PBS to remove unbound cells, fixed, stained, and quantified as previously described (15). Level of inhibition was defined based on the level of IE binding in the presence of a pool of plasma samples collected from malaria-naïve adults in the USA, using the formula [100-((Ntest/Ncontrol) x 100)].

### Flow cytometry

Mature IE described above were incubated for 30 min with plasma samples diluted 1:10, followed by incubation with anti-human IgG conjugated to phycoerythrin (PE) (eBioscience/ThermoFisher Scientific) and stained with SYBR green. A negative control included a pool of plasma samples collected from malaria-naïve adults in the USA. Reactivity of antibodies with the IE surface was expressed as percentage of IgG-stained IEs after subtracting background reactivity with the negative control. A total of 31 parasite isolates were used in the assays.

### Clinical definitions

Malaria infection was defined as the presence of parasite detected by blood smear microscopy or by PCR in blood samples collected at enrollment, gestational week 30-32 and at delivery. At delivery, both maternal peripheral blood and placental blood were examined. In addition to these fixed time points, bloodsmear analysis was performed during sick visits.

Poor pregnancy outcomes analyzed here was small for gestational age (SGA). SGA was defined according to INTERGROWTH-21 as weight below the 10^th^ percentile for gestational age (16).

### Statistical analysis

To define the plateau, a nonlinear least squares method was applied to the data via a nonlinear parameterization which estimates 3 parameters: (1) the intercept, (2) slope of the increasing line, and (3) the changepoint which determines the value of the plateau and where the plateau starts. This analysis was performed for all samples combined, and separately for women that were infected or uninfected during pregnancy.

To relate percent inhibition, breadth of inhibition and reactivity with IE surface to pregnancy outcomes, Firth’s bias-reduced logistic regression models were fitted via penalized maximum likelihood with log gestational age as an offset. This model best fitted the data because it allows to include zero counts. Median anti-adhesion levels and surface reactivity were used to avoid multiple rows per subject. Models were adjusted for the number of previous pregnancies (gravidity), infection during pregnancy up to and including delivery as a binary variable, and total number of IPTp doses received.

## Results

### Study population

Plasma samples used in this study were collected at delivery from women that participated in a longitudinal birth cohort of mother-infant pairs (6), and selected to provide a similar distribution of gravidities as seen in the full cohort (**Tables 1**, **Supplementary Table S1**). Previous studies observed increased IE reactivity in plasma collected during acute malaria infections (11), this increase could be due to boosting of existing antibodies or non-specific reactivity, therefore preference was given to samples from women that were not infected at delivery (217/232). Most women were infected at least once during pregnancy based on positive blood smear or PCR, with primigravidae experiencing more infections than other women (**Tables 1**, **Supplementary Table S1**). Overall, 71.6% of women received 2 or more IPTp-SP doses and there were no differences between gravidity groups in the number of IPTp-SP doses (p=0.3).

**Table 1:** Study population.

Group	Infection during pregnancy^a^	N anti-adhesion	N IE surface
Primigravid N=35	No infection	8	7
	>=1 infection	27	18
Secundigravid N=27	No infection	3	1
	>=1 infection	24	9
Multigravid N=170	No infection	28	20
	>=1 infection	142	106
Pregnancy outcome			
Primigravid	Early neonatal death	1	1
	PTD	2	1
	SGA	5	5
Secundigravid	PTD	2	1
	SGA	3	0
Multigravid	Miscarriage	2	2
	Stillbirth	8	6
	Early neonatal death	2	1
	PTD	10	8
	SGA	11	9

a Infection between enrollment up to and including delivery. Infections were detected by blood smear microscopy or by PCR.

PTD Preterm delivery, SGA small for gestational age.

### Anti-adhesion and IE surface-reactive antibodies progressively increase over the first 5 pregnancies

Anti-adhesion activity and IE surface reactivity were measured using fresh parasite isolates collected from pregnant women. All parasite isolates bound to the placental receptor CSA. The level and breadth of anti-adhesion activity and IE surface reactivity were analyzed in relation to the number of previous pregnancies. Delivery plasma samples and fresh heterologous parasite isolates were collected from malaria-infected pregnant women residing at the same study site. Plasma samples were used to measure anti-adhesion activity with a mean (SD) of 3.6 (1.8) isolates (range 1-10), and surface IE reactivity with 2.7 (1.3) isolates (range 1-9). Detailed number of parasite isolates analyzed in each group is described in **Supplementary Tables S2** and S3.

**Figure 1:**
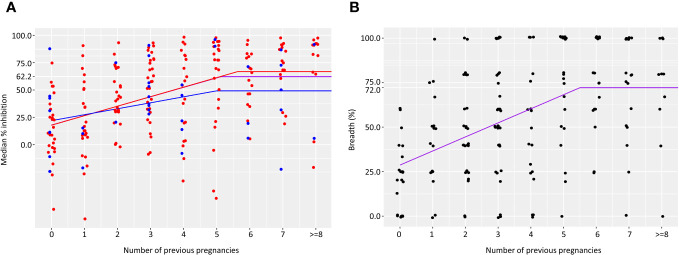
Acquisition of anti-adhesion antibodies over successive pregnancies. Median anti-adhesion levels of IE binding to CSA **(A)** was measured as the percent inhibition of binding in the presence of maternal plasma compared to the number of bound IE in the presence of naїve plasma. Number of samples in each group is as follows: 35, 27, 29, 37, 25, 25, 21, 21, 12. Purple lines represents results for all women. Results for women with no infections are shown in the blue and for women that were infected at least once in red. Breadth of anti-adhesion activity **(B)** was calculated as the percent of clinical parasite isolates in which binding was inhibited at a level of 43.7%. Number of samples in each group is as follows: 21, 17, 25, 34, 17, 21, 15, 18, 10.

We assessed the gravidity at which anti-adhesion activity and breadth of anti-adhesion activity plateau. In primigravidae, the median level of anti-adhesion activity at delivery was 17.9%. Anti-adhesion activity increased by 8.6% for each additional pregnancy up to and including the 5^th^ pregnancy. Median anti-adhesion activity in women with 5 previous pregnancies was 60.9%, and those with 6 pregnancies or more, the median anti-adhesion activity plateaued at 62.1% (**Figure 1A**). We then examined whether malaria infections detected by microscopy or by PCR during pregnancy modify anti-adhesion activity measured at delivery. There were no significant differences in the slope or the plateau between women that were or were not infected during pregnancy (**Figure 1A**), likely owing to the high infection rate prior to delivery (**Table 1**).To measure the breadth of anti-adhesion activity over successive pregnancies, multiple cutoff levels of anti-adhesion were applied based on the analysis of anti-adhesion activity for women with 2-6 previous pregnancies. Only plasma samples tested with 2 or more clinical parasite isolates were included in the breadth analysis. **Table 2** summarizes breadth analysis at different anti-adhesion cutoff values. For example, at a cutoff value of anti-adhesion activity of 43.7%, the average breadth in primigravidae was 28.7% and increased by 7.9% for each additional pregnancy up to and including the 5^th^ pregnancy. In women with 5 previous pregnancies, 68.2% of parasite isolates were inhibited at a level of 43.7% or more, and in women with 6 or more pregnancies, the breadth of activity plateaued at 72.2% (**Table 2**, **Figure 1B**).

**Table 2:** Breadth of anti-adhesion activity.

	Cutoff anti-adhesion activity level				
	35.1%	43.7%	52.3	60.9	62.1
Breadth in primigravid (%)	36.5	28.7	23.4	20.2	20
Increase per pregnancy (%)	6.7	7.9	8	7.8	7.7
Breadth: 5 previous pregnancies (%)	70	68.2	63.4	59.2	58.5
Plateau: 6 or more pregnancies (%)	76	72.2	65	60.8	60.8

Cutoff anti-adhesion activity levels are based on levels calculated for women with 2-6 previous pregnancies described in **Figure 1A** (2 previous pregnancies, 35.1%; 3 previous pregnancies, 43.7%; 4 previous pregnancies, 52.3%; 5 previous pregnancies, 60.9%; 6 previous pregnancies, 62.1%).

Similarly, we evaluated the relationship between number of previous pregnancies and level of IE surface reactivity.

**Figure 2:**
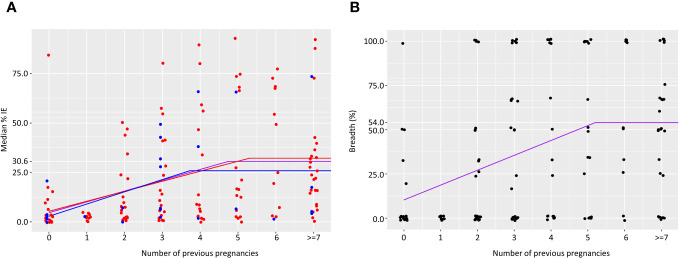
Acquisition of antibodies to surface IE over successive pregnancies. Median percent IE recognized by plasma samples **(A)**. Number of samples in each group is as follows: 25, 10, 22, 26, 18, 20, 13, 27. Purple line represents results for all women. Results for women with no infections are shown in blue and for women that were infected at least once in red. Breadth of IE surface recognition **(B)** was calculated as the percentage of clinical parasite isolates in which 21.1% or more of IEs were recognized by the plasma sample. Number of samples in each group is as follows: 17, 10, 22, 26, 17, 18, 11, 25.

In primigravidae, median level of IE surface recognition at delivery was 4.6%. Median percent of IE recognized by plasma increased by 5.5% for each additional pregnancy up to and including 4 previous pregnancies. Accordingly, the percentage of IE reacting with plasma collected from women with 4 previous pregnancies was 26.6%; in women with 5 pregnancies or more, the median IE surface reactivity plateaued at 30.5% (**Figure 2A**). IE surface reactivity correlated with anti-adhesion activity levels, r=0.53, p<0.0001. To examine the relationship between number of previous pregnancies and breadth of IE surface recognition, multiple cutoff levels of percent IE recognized were applied based on the analysis of surface reactivity over different numbers of previous pregnancies. Only plasma samples tested with 2 or more clinical parasite isolates were included in the breadth analysis. **Table 3** summarizes breadth analysis at different cutoff values for women with 2-5 previous pregnancies. For example, at a cutoff value of 21.1% of IE recognized, the average breadth in primigravidae was 10.3% and increased by 8.4% for each additional pregnancy up to and including the 5^th^ pregnancy. In women with 5 previous pregnancies, 52.3% of parasite isolates were recognized by plasma at a level of 22.1% positive IEs or more; in women with 6 or more pregnancies, the breadth of activity plateaued at 54% (**Table 3**, **Figure 2B**).

**Table 3:** Breadth of IE surface recognition.

	Cutoff level: % IE recognized			
	15.6%	22.1%	26.6%	32.1%
Breadth in primigravid (%)	12.9	10.3	9.6	7.7
Increase per pregnancy (%)	8.8	8.4	7.6	7.0
Breadth: 5 previous pregnancies (%)	56.9	52.3	47.6	42.7
Plateau: 6 or more pregnancies (%)	58.7	54	49.9	44.1

Cutoff of surface reactivity (% parasite recognized) are based on levels calculated for women with 2-5 previous pregnancies described in **Figure 2** (2 previous pregnancies, 15.6%; 3 previous pregnancies, 21.1%; 4 previous pregnancies, 26.6%; 5 previous pregnancies, 32.1%).

### Anti-adhesion activity is associated with reduced proportion of SGA births

We examined whether level and breadth anti-adhesion activity are associated with reduction in SGA births. Similar to the full cohort (8.9% of deliveries), SGA was the most common adverse pregnancy outcome (19/232 (8.2%)). Previous studies reported that malaria infection during pregnancy increased the odds of SGA (2, 17, 18), and SGA increased the risk of death during neonatal and infancy periods (19). Other adverse pregnancy outcomes were not evaluated due to low number of cases in this sample subset (**Table 1**).

Logistic regression models were fitted to examine whether increasing levels of anti-adhesion activity and breadth of anti-adhesion activity reduce SGA. The model was adjusted for the number of previous pregnancies, infection during pregnancy and number of IPTp-SP doses (**Table 4**). An increase of 10% in median anti-adhesion activity reduced the odds of SGA by 13%, and this relationship approached significance. Based on this model, an increase of 22.3% in anti-adhesion activity was expected to confer 25% reduction in the odds of SGA.

**Table 4:** Anti-adhesion activity reduces the odds of small for gestational age.

A. Anti-adhesion level			
Factor	Coefficient (95% CI)	P value	
Anti-adhesion activity	-0.013 (-0.027 – 0.001)	0.07	
Number of previous pregnancies	0.024 (-0.192 – 0.222)	0.8	
Malaria infection during pregnancy	0.804 (-0.355 – 1.850)	0.2	
IPTp doses	-0.063 (-0.878 - 0.842)	0.8	

B. Breadth of anti-adhesion activity			
Anti-adhesion cutoff level	Factor	Coefficient (95% CI)	P value
35.1%	Breadth anti-adhesion activity	-1.65 (-3.52-0.19)	0.08
	Number of previous pregnancies	0.12 (-0.12-0.34)	0.3
	Malaria infection during pregnancy	1.67 (-0.43-6.53)	0.1
	IPTp doses	0.05 (-0.83-1.05)	0.9
43.7%	Breadth anti-adhesion activity	-2.42 (-4.48- -0.52)	0.01
	Number of previous pregnancies	0.16 (-0.08-0.39)	0.2
	Malaria infection during pregnancy	1.62 (-0.48-6.49)	0.2
	IPTp doses	0.04 (-0.87-1.08)	0.9
52.3%	Breadth anti-adhesion activity	-2.22 (-4.35- -0.25)	0.03
	Number of previous pregnancies	0.15 (-0.09-0.38)	0.2
	Malaria infection during pregnancy	1.67 (-0.42-6.54)	0.1
	IPTp doses	0.03 (-0.86-1.06)	0.9
60.9%	Breadth anti-adhesion activity	-2.06 (-4.22- -0.06)	0.04
	Number of previous pregnancies	0.14 (-0.10-3.70)	0.3
	Malaria infection during pregnancy	1.71 (-0.39-6.58)	0.1
	IPTp doses	0.07 (-0.80-1.08)	0.9
62.1%	Breadth anti-adhesion activity	-2.00 (-4.15- -0.009)	0.049
	Number of previous pregnancies	0.14 (-0.10-0.37)	0.3
	Malaria infection during pregnancy	1.70 (-0.39-6.57)	0.1
	IPTp doses	0.07 (-0.80-1.08)	0.9

The associations between the breadth of anti-adhesion activity and reduction of SGA were analyzed using cutoff levels of anti-adhesion activity described in **Figure 1A**.

We evaluated the associations between the breadth of anti-adhesion activity and reduction in SGA using several cutoff levels of anti-adhesion activity, based on the analysis described in the previous section. According to the coefficient values (**Table 4B**), an increase of 10% in the breadth of anti-adhesion activity reduced the odds of SGA by 15.3%, 21.5%, 19.9%, 18.6% and 18.2% for cutoff points of anti-adhesion levels of 35.1%, 43.7%, 52.3%, 60.9% and 62.1%, respectively. After adjusting for multiple cutoff points, the relationships remained significant for a cutoff value of 43.7%.

Levels of antibodies to the IE surface and the breadth of reactivity with IE surface did not predict a reduction in odds of SGA (**Table 5**).

**Table 5:** Relationships between antibodies to IE surface and small for gestational age.

A. IE surface recognition (%)			
Factor	Coefficient (95% CI)	P value	
Median antibody reactivity	0.002 (-0.024 – 0.024)	0.9	
Number of previous pregnancies	-0.066 (-0.313 – 0.155)	0.6	
Malaria infection during pregnancy	0.172 (-1.256 – 1.369)	0.8	
IPTp doses	0.226 (-0.690 – 1.252)	0.6	

B. Breadth of IE surface recognition			
IE surface reactivity	Factor	Coefficient (95% CI)	P value
15.6%	Breadth IE surface reactivity	-0.83 (-2.76-0.83)	0.3
	Number of previous pregnancies	0.11 (-0.16-0.37)	0.4
	Malaria infection during pregnancy	1.44 (-0.67-6.31)	0.2
	IPTp doses	0.20 (-0.75-1.30)	0.7
21.1%	Breadth IE surface reactivity	-0.49 (-2.36-1.15)	0.6
	Number of previous pregnancies	0.09 (-0.17-0.34)	0.5
	Malaria infection during pregnancy	1.44 (-0.67-6.30)	0.2
	IPTp doses	0.18 (-0.77-1.29)	0.7
26.6%	Breadth IE surface reactivity	-0.72 (-2.69-0.92)	0.6
	Number of previous pregnancies	0.09 (-0.16-0.34)	0.5
	Malaria infection during pregnancy	1.43 (-0.68-6.30)	0.2
	IPTp doses	0.18 (-0.77-1.29)	0.7
32.1%	Breadth IE surface reactivity	-0.58 (-2.61-1.03)	0.5
	Number of previous pregnancies	0.09 (-0.17-0.33)	0.5
	Malaria infection during pregnancy	1.42 (-0.69-6.29)	0.2
	IPTp doses	0.17 (-0.78-1.27)	0.7

The associations between the breadth of surface reactivity and reduction of SGA were analyzed using cutoff levels of surface reactivity described in **Figure 2A**.

## Discussion

Pregnancy malaria increases the risk of adverse outcomes for both mothers and their newborn children, and existing tools to reduce such outcomes have limitations. According to WHO, only 35% of women received 3 doses of IPTp-SP (20) despite WHO recommendation more than 2 decades ago. Based on a longitudinal cohort study of pregnant women, IPTp-SP reduced the risk of adverse pregnancy outcomes during the 3 weeks that followed treatment (6). Thus, monthly doses are needed to provide protection. However, even with monthly doses, IPTp-SP cannot be used during the first trimester of pregnancy, and SP-resistant *P. falciparum* are widespread in some areas in sub-Saharan Africa, leaving women unprotected.

Therefore, a vaccine that mimics naturally acquired immunity and can be given at any time before pregnancy is needed. One of the major challenges in developing a PM vaccine has been designing a vaccine that can elicit heterologous activity like naturally acquired antibodies recognizing numerous VAR2CSA allelic forms (14). In the current study, we re-evaluated acquisition of immunity to placental malaria over successive pregnancies in order to define the level of heterologous activity that may be required for an effective vaccine.

Although women in Mali reach the plateau of anti-adhesion activity after 5 pregnancies, the benefit of anti-adhesion activity is observed at lower levels than previously reported (12). A 10% increase in anti-adhesion activity is associated with a reduction of 13% in the odds of SGA, and this relationship approached significance. An increase of 10% in the breadth of anti-adhesion significantly reduced the odds of SGA by 21.5% using a cutoff point of 43.7% anti-adhesion activity. This observation is consistent with a previous report from Benin associating anti-adhesion activity with reduced odds of SGA (21). Surface IE reactivity was not associated with a reduction in SGA. One study limitation was the low incidence of other adverse pregnancy outcomes associated with PM such as preterm delivery and stillbirth, precluded analysis. The other potential limitation is that plasma samples were used at different concentrations in the anti-adhesion activity and surface reactivity assays.

Previously, we reported that IPTp-SP significantly reduced poor pregnancy outcomes during the 3 weeks after the drug was administered (6). However, here the number of IPTp-SP doses was not associated with a reduction in SGA. It is possible that total number of IPTp doses by itself is insufficient to detect an effect unlike a time-dependent analysis that better describes the impact of the prevention strategy.

Here, both the level and the breadth of anti-adhesion activity plateaued after 5 pregnancies. In our earlier study conducted in Kenya, we reported median levels of anti-adhesion activity in secundigravid and multigravid women at delivery were 71.8% and 80.2%, respectively (13). These levels are higher than those observed in the current study. Generally, malaria cases in the whole population decreased since 2000, with the exception of an increase in cases during 2019-2020 attributed to disruption in prevention programs due to the COVID-19 pandemic (20). However, in this cohort, 73.3%, 67.1% and 64.7% of primigravid, secundigravid and multigravida women, respectively, experienced at least one malaria infection during pregnancy (6), thus reduction in malaria prevalence cannot explain the longer time to reach similar levels of anti-adhesion antibodies. Malian pregnant women participating in the current longitudinal cohort received anti-malarial treatment for infections and most women received at least 2 doses of IPTp-SP, while Kenyan pregnant women were enrolled at delivery prior to implementing IPTp-SP. It is possible that Kenyan pregnant women were exposed to placental parasite proteins for a longer period resulting in higher anti-adhesion activity. Although it appears that in the Malian population it takes longer to reach plateau levels of anti-adhesion activity and the breadth of anti-activity, the benefit of anti-adhesion antibodies was observed at antibody levels below the maximal anti-adhesion activity.

Levels of antibodies that recognize IE surface and the breadth of surface IE recognition also increase during the first 4-5 pregnancies. The increase of median antibody levels over the first 4 pregnancies is similar to a previous study describing increased antibodies reacting to the surface of CSA-selected IEs with increasing number of previous pregnancies (22, 23). Unlike anti-adhesion activity, IE surface reactivity was not associated with a reduction in SGA in this study, similar to previous studies reporting non-significant reduction or no change in the odds of LBW in women with antibodies with IE surface reactivity (21, 24).

The relationships between antibodies to recombinant VAR2CSA domains or full-length protein vary between studies, with some studies reporting a relationship between antibodies to some VAR2CSA domains and reduction in LBW (summarized in (24)). Two studies evaluated the association between antibodies to recombinant VAR2CSA domains or full-length protein and SGA. In Benin, increasing levels of antibodies to DBL3 domain at enrollment (but not afterwards) were associated with a trend of reduced SGA (21). In the study in Mali, antibodies to VAR2CSA measured during pregnancy did not predict a reduction in SGA and similarly, antibody levels at delivery were not associated with reduced odds of SGA (25). In the current study conducted in Mali as well as the study in Benin, the presence of anti-adhesion antibodies was significantly associated with reduced odds of SGA (21), suggesting antibodies to recombinant proteins or reactivity with IE surface are qualitatively different than functional antibodies that reduce parasite adhesion.

The finding in this study could guide the development of a PM vaccine. The study showed that the breadth of anti-adhesion is associated with a significant reduction in SGA even at a cutoff point of 43.7% anti-adhesion activity. Thus, the breadth of activity appears to be an important factor rather than the level of anti-adhesion by itself. As such, high levels of anti-adhesion activity to a limited VAR2CSA allelic forms may be less protective than a vaccine eliciting lower anti-adhesion activity but with a larger breadth.

In summary, we described that anti-adhesion antibody levels plateaued after several pregnancies. An increase in anti-adhesion activity and the breadth of activity are associated with a reduction in SGA births. This information can guide evaluation of PM vaccine candidates by providing benchmark indicators that will predict impact on poor birth outcomes.

## Data availability statement

The original contributions presented in the study are included in the article/**Supplementary Material**. Further inquiries can be directed to the corresponding author.

## Ethics statement

The studies protocols and procedures involving humans were approved by the Institutional Review Boards at the National Institute of Allergy and Infectious Diseases, National Institutes of Health, and by the Ethics Committee of the Faculty of Medicine, Pharmacy and Dentistry, University of Bamako. The studies were conducted in accordance with the local legislation and institutional requirements. The participants provided their written informed consent to participate in this study.

## Author contributions

AM: Investigation, Project administration, Writing–review & editing. BS: Formal analysis, Visualization, Writing–review & editing. MT, OA, BSD, GS, DI, AB, YS, YTD, SK and ON: Investigation, Writing–review & editing. AD: Conceptualization, Supervision, Writing–review & editing. PD: Conceptualization, Supervision, Writing–review & editing. MF: Conceptualization, Supervision, Writing–original drafts, Writing-review & editing.
