# Supplementary material for: Acquisition of antibodies that block Plasmodium falciparum adhesion to placental receptor chondroitin sulfate A with increasing gravidity in Malian women
Source: Front Immunol. 2024 Jan 11;14:1330962. doi: 10.3389/fimmu.2023.1330962 (PMC10808177; doi:10.3389/fimmu.2023.1330962)
Supplement: Supplementary file 1 [file Table_1.docx]

**Supplementary Table S1. Multigravid women stratified by the number of previous pregnancies**

| Number of previous pregnancies (n) | No infection (n) | >=1 infection^a^ (n) | Pregnancy loss (n) | PTD (n) | SGA (n) |
| --- | --- | --- | --- | --- | --- |
| 2 (29) | 3 | 26 | 1 | 0 | 1 |
| 3 (37) | 8 | 29 | 3 | 2 | 1 |
| 4 (25) | 5 | 20 | 2 | 0 | 3 |
| 5 (25) | 2 | 23 | 3 | 1 | 2 |
| 6 (21) | 4 | 17 | 2 | 1 | 1 |
| 7 (21) | 6 | 15 | 1 | 2 | 1 |
| >=8 (12) | 0 | 12 | 0 | 4 | 2 |

^a^ Infection between enrollment up to and including delivery. Infection was detected by blood smear microscopy or by PCR.

Pregnancy loss includes miscarriage, stillbirth, and early neonatal death. PTD Preterm delivery, SGA small for gestational age.

**Supplementary Table S2. Number of parasite isolates used in anti-adhesion assays by the number of previous pregnancies**

| Number of previous pregnancies | N | Mean number of parasite isolates | Range of parasite isolates |
| --- | --- | --- | --- |
| 0 | 35 | 3.3 | 1-10 |
| 1 | 27 | 2.7 | 1-5 |
| 2 | 29 | 3.8 | 1-5 |
| 3 | 37 | 3.9 | 1-5 |
| 4 | 25 | 3.5 | 1-5 |
| 5 | 25 | 3.8 | 1-5 |
| 6 | 21 | 3.3 | 1-5 |
| 7 | 21 | 3.8 | 1-5 |
| >=8 | 12 | 4.4 | 1-9 |

**Supplementary Table S3. Number of parasite isolates used in surface reactivity assays by the number of previous pregnancies**

| Number of previous pregnancies | N | Mean number of parasite isolates | Range of parasite isolates |
| --- | --- | --- | --- |
| 0 | 25 | 2.6 | 1-7 |
| 1 | 10 | 2.6 | 2-4 |
| 2 | 22 | 2.5 | 2-4 |
| 3 | 26 | 2.7 | 2-9 |
| 4 | 18 | 2.6 | 1-6 |
| 5 | 20 | 2.5 | 1-5 |
| 6 | 13 | 2.5 | 1-4 |
| 7 | 17 | 3.4 | 1-9 |
| >=8 | 10 | 3.3 | 3-4 |
